# Autoantibodies to Endothelial Cell Surface ATP Synthase, the Endogenous Receptor for Hsp60, Might Play a Pathogenic Role in Vasculatides

**DOI:** 10.1371/journal.pone.0014654

**Published:** 2011-02-07

**Authors:** Jean-Eric Alard, Sophie Hillion, Loïc Guillevin, Alain Saraux, Jacques-Olivier Pers, Pierre Youinou, Christophe Jamin

**Affiliations:** 1 EA2216 “Immunology and Pathology” and IFR 148 ScInBioS, Université de Brest and Université Européenne de Bretagne, Brest, France; 2 Department of Internal Medicine, Hôpital Cochin, Paris, France; 3 Centre Hospitalier Universitaire, Brest, France; University of Sheffield, United Kingdom

## Abstract

**Background:**

Heat shock protein (hsp) 60 that provides “danger signal” binds to the surface of resting endothelial cells (EC) but its receptor has not yet been characterized. In mitochondria, hsp60 specifically associates with adenosine triphosphate (ATP) synthase. We therefore examined the possible interaction between hsp60 and ATP synthase on EC surface.

**Methodology/Principal Findings:**

Using Far Western blot approach, co-immunoprecipitation studies and surface plasmon resonance analyses, we demonstrated that hsp60 binds to the β-subunit of ATP synthase. As a cell surface-expressed molecule, ATP synthase is potentially targeted by anti-EC-antibodies (AECAs) found in the sera of patients suffering vasculitides. Based on enzyme-linked immunosorbent assay and Western blotting techniques with F1-ATP synthase as substrate, we established the presence of anti-ATP synthase antibodies at higher frequency in patients with primary vasculitides (group I) compared with secondary vasculitides (group II). Anti-ATP synthase reactivity from group I patients was restricted to the β-subunit of ATP synthase, whereas those from group II was directed to the α-, β- and γ-subunits. Cell surface ATP synthase regulates intracellular pH (pHi). In low extracellular pH medium, we detected abnormal decreased of EC pHi in the presence of anti-ATP synthase antibodies, irrespective of their fine reactivities. Interestingly, soluble hsp60 abrogated the anti-ATP synthase-induced pHi down-regulation.

**Conclusions/Significance:**

Our results indicate that ATP synthase is targeted by AECAs on the surface of EC that induce intracellular acidification. Such pathogenic effect in vasculitides can be modulated by hsp60 binding on ATP synthase which preserves ATP synthase activity.

## Introduction

Adenosine triphosphate (ATP) synthase, or F0F1-ATPase, produces and hydrolyzes ATP with proton translocation [1]. F0 operates as a proton channel with a rotation driving the F1 to synthesize ATP, depending on the direction of rotation. F1 comprises 3 β-subunits assuming the catalytic activity modulated by 3 α-subunits alternately ordered to form a cylinder, completed by a γ-subunit located at the center of the αβ stalk, that constitutes the key rotary element in the enzyme's catalytic activity [2].

ATP synthase is resident in the inner mitochondrial membrane. However, evidence suggests that it is also localized in cell membranes, and translocate into the lipid rafts (LRs) of normal endothelial cells (EC). Depending on cell type [3], cell surface ATP synthase triggers hydrolysis or synthesis of ATP, modulates angiogenesis, cellular immunity, cholesterol uptake and regulates intracellular pH (pHi).

Cell surface ATP synthase acts also to bind several ligands and to control EC proliferation and differentiation [4]. For example, angiostatin binds to αβ-subunits, blocks ATP synthase activity when EC are in a low extracellular pH (pHe) environment, and is thus responsible for the inhibition of proton flux due to pH stress [5]. The overall consequence is intracellular acidification that induces EC death and inhibits neovascularisation [6]. By contrast, apoliprotein A-I stimulates F1-ATPase activity following binding and generates adenosine diphosphate that inhibits EC apoptosis and promotes proliferation [7].

Alteration in ATP synthase function could therefore cause significant damages to EC homeostasis. Furthermore, in the mitochondria, heat shock protein (hsp)60 specifically associates with ATP synthase [8], and ensures correct assembly of the complex. Hsp60 is also present on EC surface [9]. Though several ligands for different hsps have been listed [10], there is no clear evidence about the one or those which can specifically bind to hsp60 when found on the surface of EC. Thus, hsp60 binds to EC irrespective of TLR2, TLR4, CD91 or CD14 expression [11], [12]. Mitochondrial hsp70 has been identified as one ligand for hsp60 on the surface of stressed EC [13], but its receptor remains uncharacterized in non-stressed conditions. Their intra-mitochondrial association suggests that translocation into extra-mitochondrial sites might facilitate ATP synthase and hsp60 interactions. Interestingly, both ATP synthase and hsp60 can cause cytolysis [4], [14]. Hsp60 behaves as an antigenic target for antibodies (Abs), such as anti-EC Abs (AECAs) [15] which are frequently associated with vascular inflammation [16], and plays a role in promoting and regulating autoimmunity [17], [18]. Therefore, mitochondrial proteins can generate immune responses contributing to damaged EC. Their presence on the EC surface and the subsequent effects of Ab binding might participate in the pathogenesis of vasculitides. Depending on the site and the type of blood vessels affected, clinical and pathological manifestations vary considerably. This awareness has justified nomenclature of vasculitdies [19]. These diseases may be autonomous and referred to as primary vasculitides. They may affect small vessels in Wegener's granulomatosis (WG), Churg-Strauss syndrome (CSS), microscopic polyangiitis (MPA), medium vessels in polyarteritis nodosa (PAN) or large vessels. These primary forms result from vasculitis which is the triggering abnormality. Vasculitides may also be set against a background of autoimmune diseases such as systemic lupus erythematosus (SLE), primary Sjögren's syndrome (pSS), or rheumatoid arthritis (RA) and are then designated as secondary vasculitides [20]. The current work was pursued to first characterize possible interactions between ATP synthase and hsp60 on the EC membrane, and second to evaluate the existence of anti-ATP synthase Abs in vasculitides and their impact on EC.

## Results

### I – ATP synthase is a receptor for hsp60

**Figure 1:**
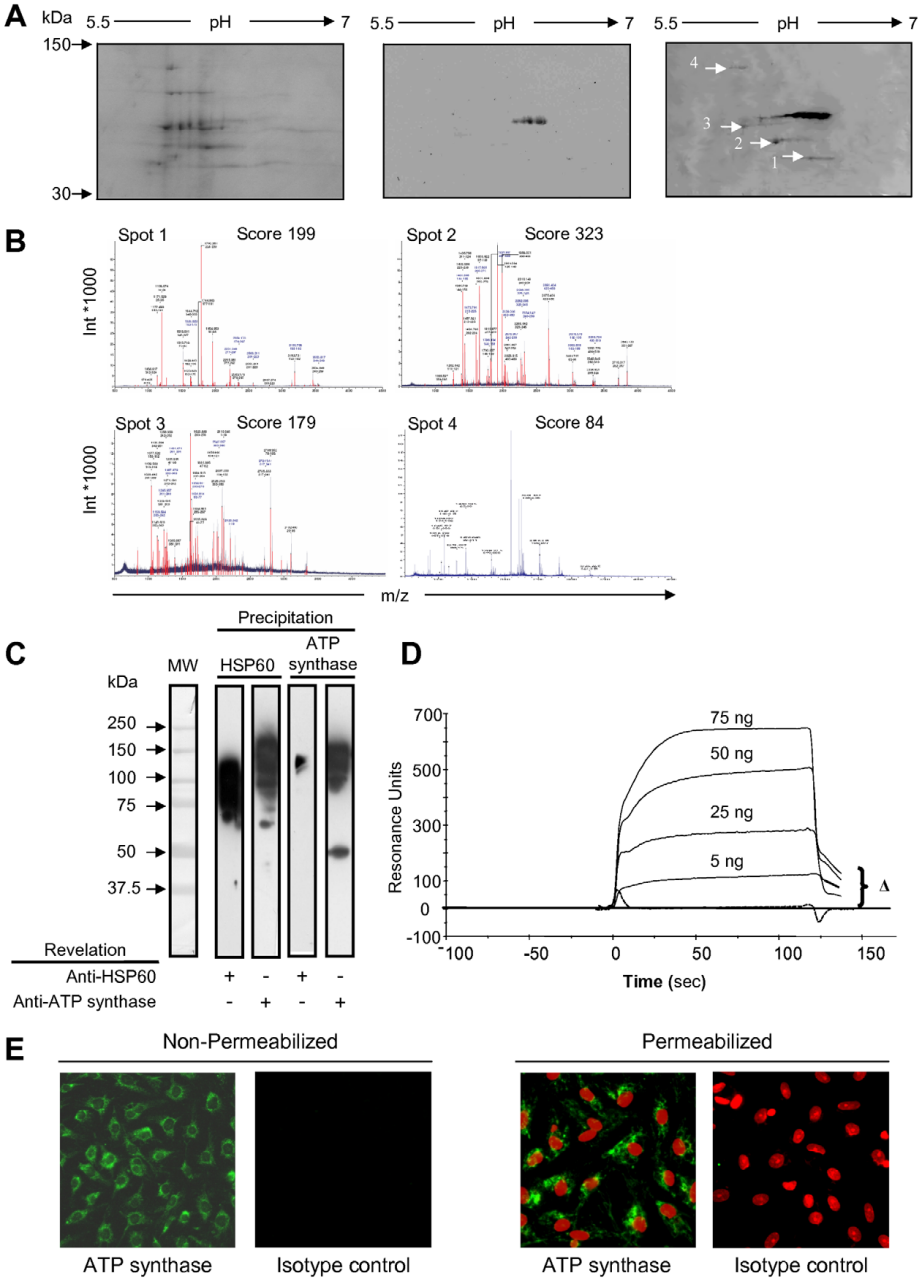
ATP synthase is a receptor for soluble hsp60. **A-** HUVEC membrane-enriched protein were electrophoresed on bidimensional gels. Left panel: staining with Coomassie blue. Middle panel: WB with anti-hsp60 mAb. Right panel: WB with recombinant hsp60 revealed with anti-hsp60 mAb. **B-** Mass spectrometry data of proteins bound to hsp60. **C**- Hsp60 and ATP synthase were co-immunoprecipitated from membrane-enriched protein extracts of EAhy926 cells with anti-ATP synthase and anti-hsp60mAbs, respectively. Proteins were analysed by WB using polyclonal anti-hsp60 or anti-ATP synthase Abs. MW markers are on the left. **D-** Increasing amounts of ATP synthase were passed over hsp60 immobilized onto a sensor chip. Sensogram of each amount of analyte with substraction of non-specific binding represent resonance units. Specific binding is shown (Δ). **E-** EAhy926 cells, permeabilized or not, were incubated with a primary anti-ATP synthase Ab revealed by FITC-conjugated anti-Ig Ab, and intracellular as well as cell-surface expression of ATP synthase analyzed by fluorescence microscopy. Propidium iodide was used to visualize nuclei, and isotype control staining performed as negative control.

A minority of resting human umbilical vein EC (HUVEC) bound to soluble hsp60 [15], suggesting that the “danger” signal provided by hsp60 [21] might be detected by an as yet unidentified receptor. To do so, we opted for a Far Western blot (WB) approach. HUVEC membrane-enriched proteins were resolved on 3 bidimensional gels. The first was stained with Coomassie blue to verify the protein extraction procedure (Figure 1A, left panel). Proteins from the second were blotted onto a PVDF membrane, and probed with anti-hsp60 monoclonal Ab (mAb) to localize the endogenous hsp60 (Figure 1A, middle panel). Proteins from the third were incubated with recombinant hsp60 and probed with anti-hsp60 mAb. This procedure identified 4 additional spots relative to the second gel (Figure 1A, right panel). They were cut out from the Coomassie blue-stained gel, analyzed by mass spectroscopy and their amino acid sequence determined after screening of the Swiss-Prot databank. Actin, ATP synthase β-chain, prolyl-4-hydroxylase β-subunit and gp96 were identified (Figure 1B). We focused on ATP synthase protein, and, to confirm hsp60/ATP synthase β-subunit interaction, performed reverse co-precipitation experiments. Immunoprecipitation with either mAbs co-precipitated the other protein as shown by the 128-kDa molecular weight (MW) complex (Figure 1C). The presence of smears around this MW suggest the persistant interactions between different ATP synthase subunits, and also that other proteins may interact with ATP synthase or with hsp60. Therefore, to demonstrate that ATP synthase could be the receptor for hsp60, recombinant hsp60 was immobilized onto the biosensor chip of a Biacore X system, ATP synthase injected into the cell flow and their interaction followed in real time (Figure 1D). Sensograms indicated that hsp60 interacts with ATP synthase as shown by the curves that did not return to baseline.

Although ATP synthase is abundantly expressed in the cytoplasm of permeabilized EC, a modest level of fluorescence was found on the surface of nonpermeabilized EC by indirect immunofluorescence (Figure 1E). This suggests that ATP synthase could be accessible to hsp60 on the EC surface.

### II – Detection of ATP synthase reactivity

**Figure 2:**
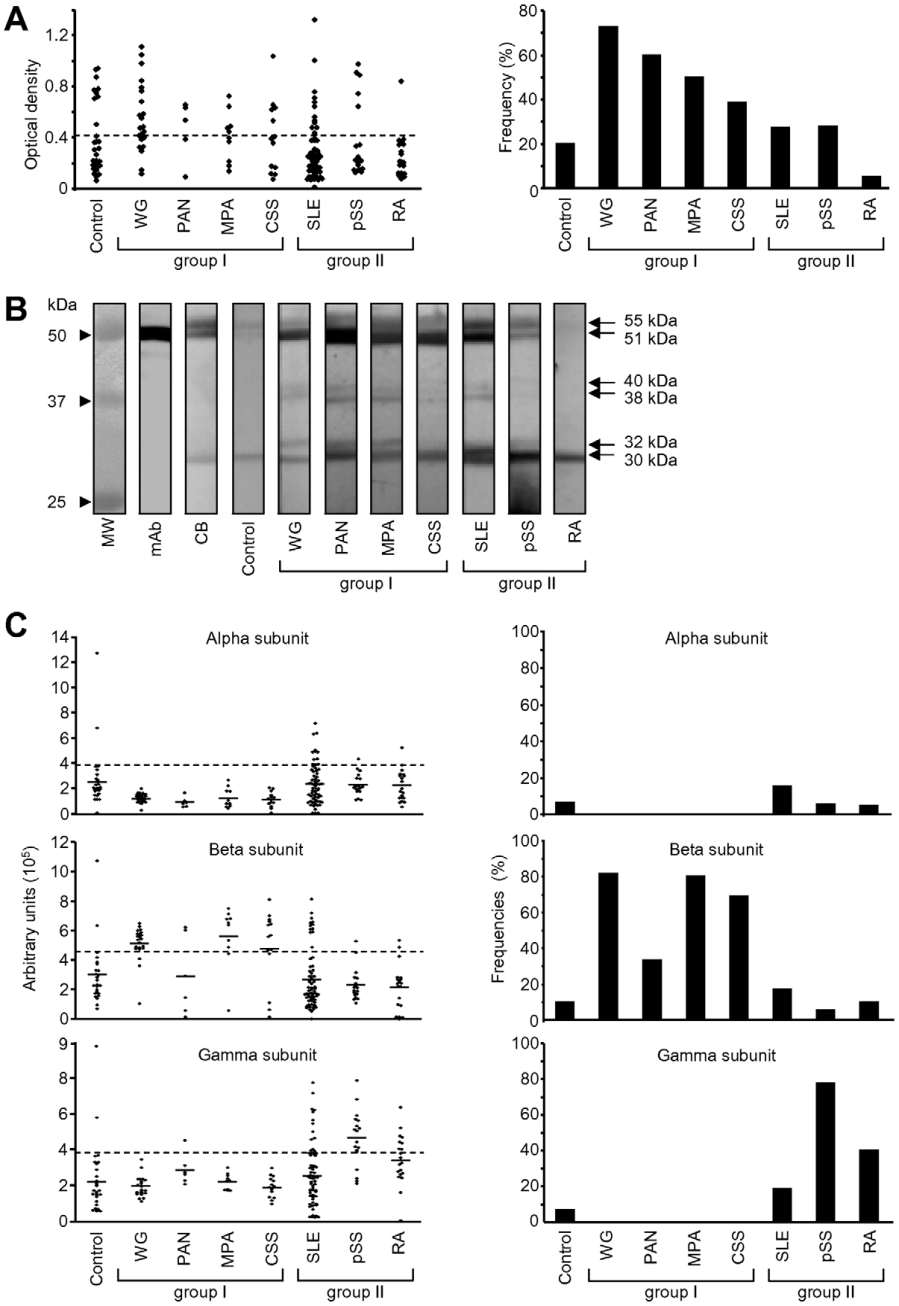
Reactivity of sera with ATP synthase. **A-** Optical densities (left panel) and percentages of positivity (right panel) of sera from patients in ELISA with purified ATP synthase. Broken line depicts the cut off level for positivity (OD 0.410, representing the mean+2 SD of control values). Group I corresponds to patients with primary vasculitides (WG, PAN, MPA and CSS) and group II to patients with secondary vasculitides associated with autoimmune diseases (SLE, pSS, RA). **B-** Purified ATP synthase was subjected to electrophoresis and blotted with sera from patients and controls. Representative results from controls, group I and group II patients are shown. MW markers are on the left. Result with anti-β-subunit mAb and Coomassie blue (CB) staining are shown as controls. **C-** Densitometry measurement (left panels) and percentages (right panels) of sera activities against α-, β- and γ-ATP synthase subunits by Western blots. Broken lines show the cut off level for positivity (OD 3.9.10^5^, 4.5.10^5^ and 3.8.10^5^ arbitrary units, representing the mean+2 SD of control values for α-, β-, and γ-subunit, respectively). WG = Wegener's granulomatosis; PAN = polyarteritis nodosa; MPA = microscopic polyangiitis; CSS = Churg-Strauss syndrome; SLE = systemic lupus erythematosus; pSS = primary Sjögren's syndrome; RA = rheumatoid arthritis.

We have previously demonstrated that among the EC surface-expressed molecules [16] hsp60 could be a target for AECAs [15]. To assess whether ATP synthase, receptor for hsp60 was recognized by AECAs as well, we screened AECA-positive sera from different patient groups suffering vasculitides with an in-house enzyme-linked immunosorbent assay (ELISA) using bovine F1-ATP synthase as substrate. Sera were considered positive when the optical density (OD) value was over than 0.410 (Figure 2A, left panel). Thus, as shown in right panel of Figure 2A, 20% of controls were reactive and there were more sera from group I patients suffering primary vasculitides (36–73%) positive for ATP synthase binding than from group II patients suffering secondary vasculitides (5–28%, *p*<0.001).

Because a subgroup of controls displayed positivity, we asked the question as to whether ATP synthase reactivity was similar to that observed in sera from patients and whether those from group I and II were different. To answer this question, reactivities were evaluated by WB. Anti-ATP synthase Abs bound to 3 major bands with a MW of 55, 51 and 30 kDa (Figure 2B), and 3 minor bands of 40, 38 and 32 kDa, that were sequenced. The 3 major bands were the α-, β- and γ-subunits of F1-ATP synthase, and the 3 minor bands were degradation products of the β-subunit. Sera reactivities were considered positive when the OD values after densitometry analysis were over 3.9.10^5^ AU, 4.5.10^5^ AU and 3.8.10^5^ AU for α-, β-, and γ-subunit, respectively (Figure 2C, left panels). Regarding overall reactivities, more than 13% of sera from the controls were positive in the WB. Furthermore, more sera from group I were positive (50–86%) than those from group II (45–78%, *p* = 0.005). In the controls, frequencies of positive sera were 6.7, 10 and 6.7% for α-, β- and γ-subunit, respectively. (Figure 2C, right panels). Reactivity was strikingly homogeneous in sera from group I patients since all were reactive only for the β-subunit. In group II patients, percentages of positivity ranged from 5–15.6% for the α-subunit, 5.6–17.2% for the β-subunit and 18.7–77.8% for the γ-subunit.

**Figure 3:**
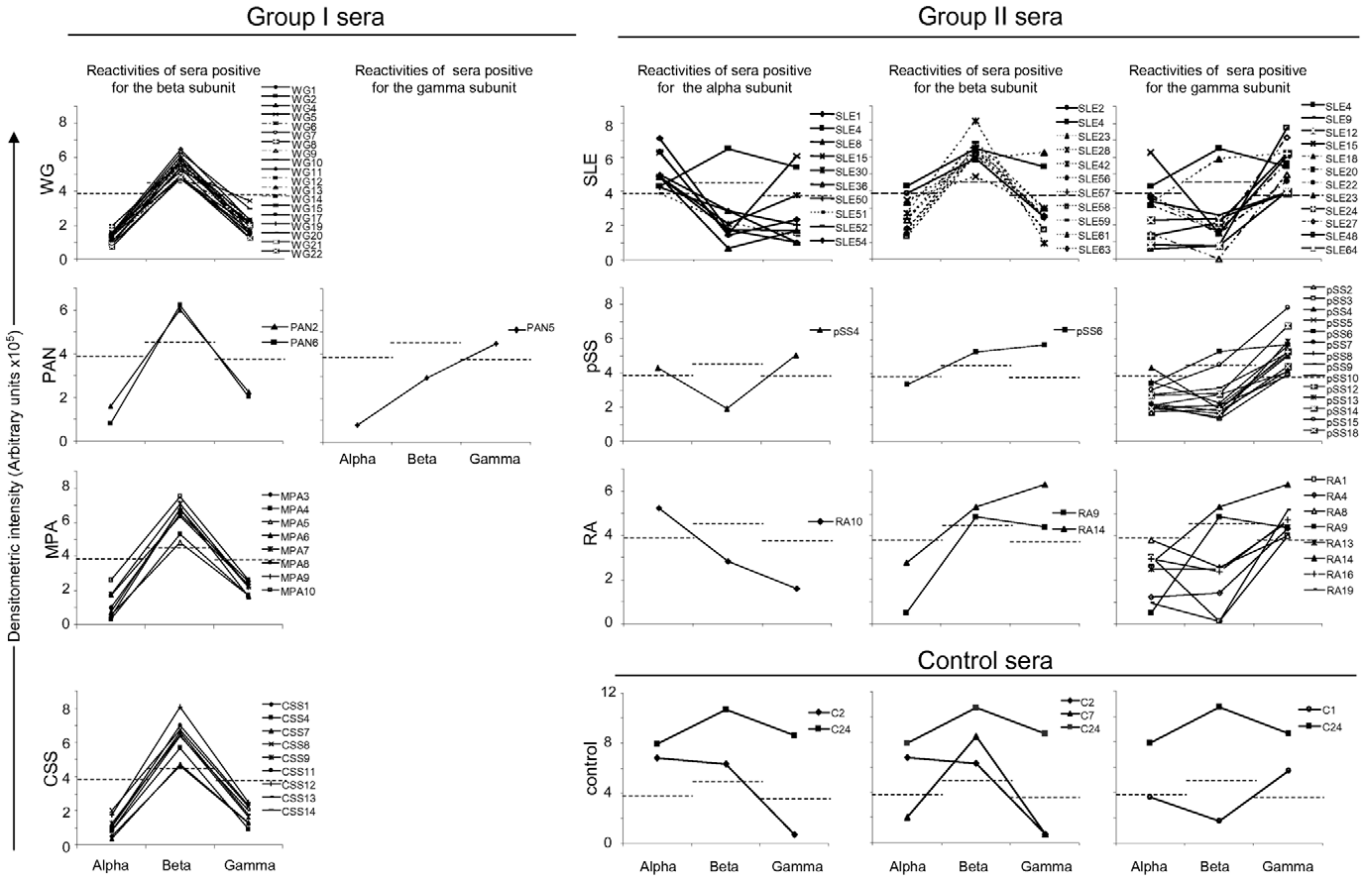
Multireactivity of anti-ATP synthase Ab positive sera. Sera from group I (WG, PAN, MPA, CSS), group II (SLE, pSS, RA) patients and controls positive for the α-, β-, or γ-subunit of ATP synthase were evaluated for their reactivity against all subunits. Densitometry measurements of reactivity by Western blots are shown. Broken lines show the cut off level for positivities against α-subunit (3.9.10^5^ AU), β-subunit (4.3.10^5^ AU) and γ-subunit (3.9.10^5^ AU), corresponding to the mean+2 SD of control values. WG = Wegener's granulomatosis; PAN = polyarteritis nodosa; MPA = microscopic polyangiitis; CSS = Churg-Strauss syndrome; SLE = systemic lupus erythematosus; pSS = primary Sjögren's syndrome; RA = rheumatoid arthritis.

We, next, assessed the latent multi-reactivity of positive anti-ATP synthase sera. Positivity for the β-subunit in group I was not associated with other reactivity (Figure 3), and the one positive for the γ-subunit was negative for the others. It is concluded that sera from patients with primary vasculitides exhibited homogeneous reactivity to F1-ATP synthase. By contrast, 1 control serum positive for α-subunit (C24) was also positive for β-, and 1 (C2) was positive for the γ-subunit as well. One control was only positive for the β-subunit (C7), and another 1 (C1) for the γ-subunit. When positive, reactivities of control sera for F1-ATP synthase appeared heterogeneous. In group II patients, 7 SLE sera were only reactive for the α-subunit (SLE1, 8, 36, 50, 51, 52 and 54), 2 (SLE15 and SLE30) were positive for the α-subunit and also for the γ-subunit, and 1 (SLE4) for the α-subunit, the γ-subunit and the β-subunit too. Eleven SLE sera were positive for the β-subunit among which 9 had no other reactivities (SLE2, 28, 42, 56, 57, 58, 59, 61 and 63) and 1 (SLE23) was also positive for the γ-subunit. Finally, 9 of 12 sera positive for the γ-subunit displayed a single reactivity (SLE9, 12, 18, 20, 22, 24, 27, 48 and 64). Reactivities of pSS patients appeared to be less heterogeneous since all positive sera reacted with the γ-subunit, among which only 1 (pSS4) was also positive for the α-subunit and one (pSS6) also positive for the β-subunit. Finally, all RA sera were positive for the γ-subunit, but 1 (RA10) reactive with the α-subunit. Two of them (RA9 and RA14) were also positive for the β-subunit. We concluded that among anti-ATP synthase reactive sera, those from patients with secondary vasculitides presented the broadest multi-reactivity pattern (*p* = 0.04) for the F1-ATP synthase subunits (Table 1).

**Table 1:** ATP synthase multi-reactivities in sera from controls and from patients with primary and secondary vasculitides associated systemic autoimmune diseases found positive by Western blotting*.

	Frequencies (no) of anti-ATP synthase reactivity						
α-subunit	+	−	−	+	−	+	+
β- subunit	−	+	−	+	+	−	+
γ-subunit	−	−	+	−	+	+	+
Control (n = 4)	0	25.0 (1)	25.0 (1)	25.0 (1)	0	0	25.0 (1)
**Primary vasculitides**							
WG (n = 19)	0	100.0 (19)	0	0	0	0	0
CSS (n = 9)	0	100.0 (9)	0	0	0	0	0
PAN (n = 3)	0	66.6 (2)	33.3 (1)	0	0	0	0
MPA (n = 8)	0	100.0 (8)	0	0	0	0	0
**Secondary vasculitides**							
SLE (n = 29)	27.6 (8)	31.1 (9)	31.1 (9)	0	3.4 (1)	3.4 (1)	3.4 (1)
pSS (n = 14)	0	0	85.8 (12)	0	7.1 (1)	7.1 (1)	0
RA (n = 9)	11.1 (1)	0	66.7 (6)	0	22.2 (2)	0	0

*ATP synthase reactivities to the 55 kDa α-subunit, the 51 kDa β-subunit and the 30 kDa γ-subunit were determined by Western blotting.

### III – Modulation of ATP synthase function

**Figure 4:**
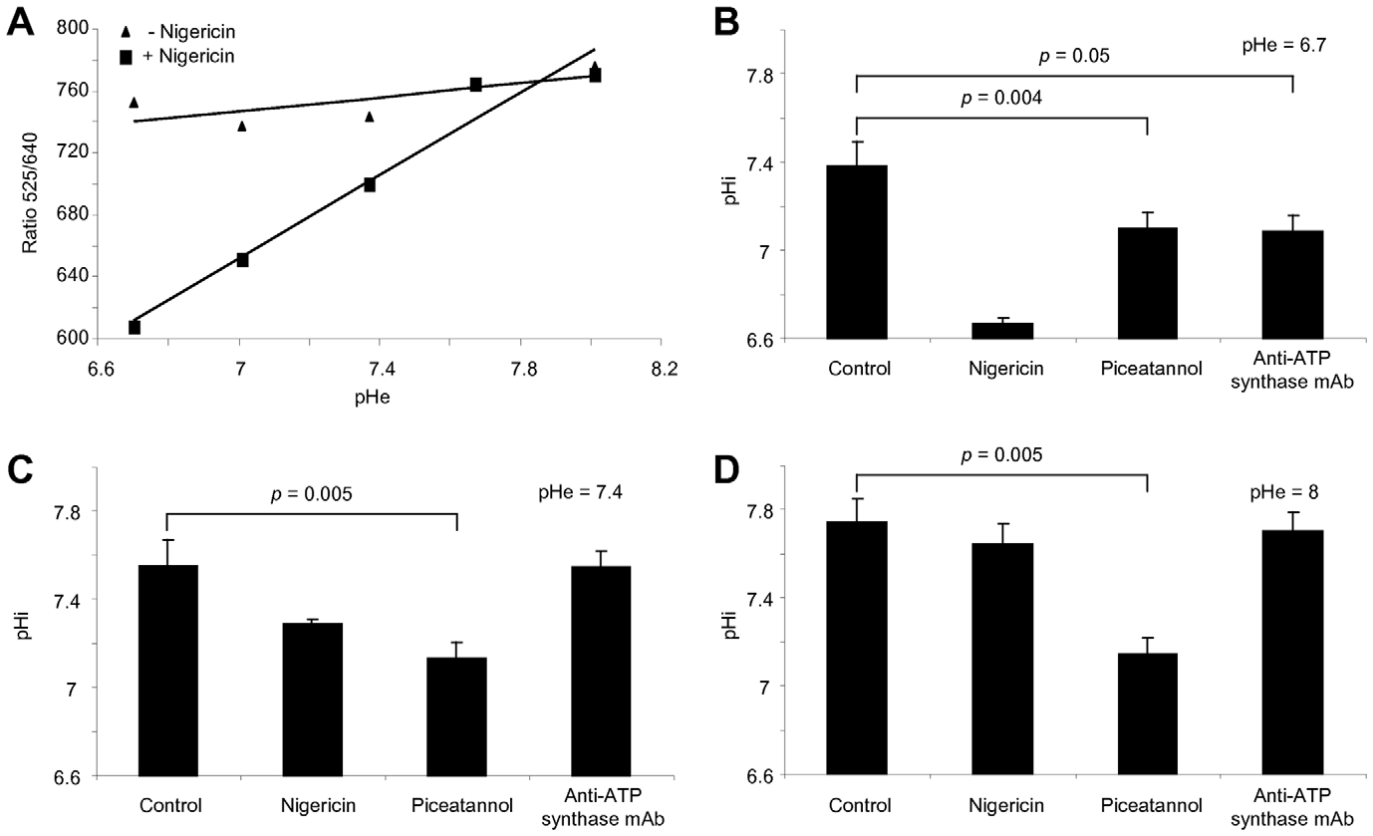
ATP synthase regulates intracellular pH. EAhy926 cells were labelled with BCECF, incubated with or without 40 µM nigericin in medium at different extracellular pH (pHe), and analyzed by flow cytometry. Fluorescence emission ratio 525nm/640nm (green fluorescence/red fluorescence) following BCECF excitation was calculated to determine calibration curves (**A**). BCECF-labelled EAhy926 cells were incubated either alone (control), with 40 µM nigericin, 80 µM piceatannol, or 50 µg/ml anti-ATP synthase mAb in medium at pHe 6.7 (**B**), 7.4 (**C**), or 8 (**D**). Ratio 525/640 was calculated and intracellular pH (pHi) determined with the calibration curves. Mean±SD of 3 experiments.

We then asked the question as to whether anti-ATP synthase Abs could alter the ATP synthase-dependent regulation of pHi [3]. EC were stained with 2′,7′-*bis*-carboxyethyl-5,6-carboxyfluorescein (BCECF), and pilot experiments elaborated by flow cytometry to validate the protocol that measures emission intensities of BCECF at 525nm and 640nm. To this end, EAhy926 EC line cells were treated with nigericin and incubated in medium with different pHe from 6.7 to 8. Nigericin is an ionophore that exchanges K^+^ and H^+^ across cellular membranes [22]. pHi measured in these conditions corresponds to pHe. Intracellular 525/640 ratios increased with pHe (Figure 4A) indicating that flow cytometry analyses were efficient. Moreover, the ratio was left stable without nigericin, demonstrating that nontreated EC retained their ability to maintain constant pHi between pHe 6.7 to 8. Additionally, to evaluate ATP synthase function, EC were pre-treated with piceatannol, an inhibitor of ATP synthase [23], and incubated in an acidic pHe 6.7 medium. pHi decreased to 7.1±0.1 while it was maintained at 7.38±0.11 (*p* = 0.004) in medium free from ATP synthase inhibitor (Figure 4B), demonstrating the importance of ATP synthase activity in pHi regulation. Interestingly, pre-treatment of EC with anti-ATP synthase mAb also reduced pHi to 7.08±0.07 (*p* = 0.005). These results indicate that anti-ATP synthase Abs can inhibit ATP synthase function. By contrast, pHi remained stable at 7.55±0.07 (*p* = 0.6, Figure 4C) and 7.7±0.09 (*p* = 0.37, Figure 4D) when EC were incubated with anti-ATP synthase mAb in neutral (pHe 7.4) or basic (pHe 8) medium, respectively. These experiments demonstrate that anti-ATP synthase mAb alters ATP synthase activity only in acidic environment. This conclusion was confirmed by pre-treatment of EC with oligomycin another inhibitor of ATP synthase [24]. pHi decreased only when EC were incubated in acidic pHe medium. Furthermore, no effects could be observed with anti-hsp Abs, with sodium azide at the concentration found in anti-ATP synthase mAb preparation, nor with DMSO (not shown).

**Figure 5:**
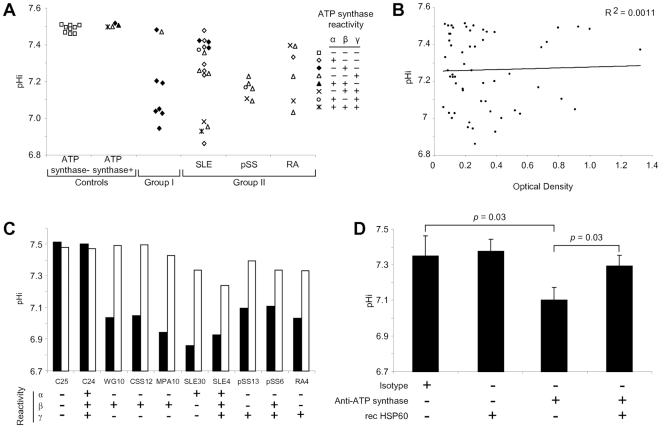
Anti-ATP synthase auto-Abs modulate ATP synthase function. **A-** EAhy926 cells were labelled with BCECF, incubated in pH 6.7 medium with sera showing different reactivity patterns against α-, β-, and γ-subunit of ATP synthase, and analyzed by flow cytometry. Intracellular pH (pHi) was then calculated from calibration curves. **B-** Correlation between ATP synthase reactivity determined in ELISA and pHi of EAhy926 cells incubated in the presence of sera in pH 6.7 medium. **C-** ATP synthase positive sera were depleted (open bars) or not (solid bars) of their ATP synthase reactivity and incubated with BCECF-loaded EAhy926 cells in pH 6.7 medium. After flow cytometry analyses, pHi were determined using calibration curves. ATP synthase positive (C24) and negative (C25) sera from healthy donors were used as controls. **D-** BCECF-loaded EAhy926 cells were incubated with recombinant hsp60 before suspension in medium pH 6.7 in the presence of 50 µg/ml anti-ATP synthase mAb. After flow cytometry analyses, pHi were determined using calibration curves. Mean±SD of 3 experiments. WG = Wegener's granulomatosis; CSS = Churg-Strauss syndrome; MPA = microscopic polyangiitis; SLE = systemic lupus erythematosus; pSS = primary Sjögren's syndrome; RA = rheumatoid arthritis.

Effect of anti-ATP synthase Abs from sera was therefore evaluated with EC incubated at pHe 6.7 (Figure 5A). Sera from controls, reactive or unreactive for ATP synthase, did not reduce pHi which were maintained at 7.45–7.51. Nearly all sera positive for ATP synthase from group I patients reduced pHi of EC between 6.93 and 7.2. Some had no effect with pHi sustained at 7.46–7.48. ATP synthase positive sera from group II patients displayed diverse effects. A striking down-regulation between 6.85–6.97 was observed with a subgroup of sera from SLE patients, indicating a strong inhibition of ATP synthase function. Another subgroup slightly diminished pHi between 7.23–7.48. Sera from patients with pSS induced intermediate inhibition of ATP synthase activity with pHi decreased between 7.08 and 7.22. Finally, sera from RA patients were variable, from a strong decrease of pHi to 7.05 to a minor reduction at 7.39. Overall, these data demonstrate varied effects of anti-ATP synthase Abs from sera on ATP synthase function.

To further elucidate this issue, we appraised specific ATP synthase subunit reactivity. Positivity for α-, β- and/or γ-subunits were inspected in sera inducing or not down-regulation of pHi. As shown in Figure 5A, effect on pHi regulation could not be designated with particular ATP synthase reactivities. Thus, while some β-reactive sera decreased pHi others had no effect. Similarly, some α-reactive sera did not affect pHi whilst others reduced it. Moreover, multireactivities did not appear as a decisive factor either. These prompted us to assess Ab titres. The analysis did not show a correlation between OD values for the ELISA and pHi values of EC determined in the presence of sera (Figure 5B), suggesting that effect on pHi was not related to Ab concentration. Nevertheless, down-regulations of pHi were abrogated when sera were depleted of their anti-ATP synthase reactivities by overnight incubation in ATP synthase-coated microplates (Figure 5C). These data confirm that effects of anti-ATP synthase positive sera on pHi modulation are specifically due to ATP synthase recognition.

### IV – Regulatory role for hsp60 in ATP synthase activity

Once hsp60 was shown to be a novel ligand for ATP synthase on EC surface, we sought to evaluate its role on the function of ATP synthase. Binding of recombinant hsp60 to EC cultured in acidic pH 6.7 medium had no effect on pHi that was retained at 7.38±0.07, compared with pHi 7.35±0.11 of control EC (*p* = 0.76, Figure 5D). This data indicates that binding of soluble hsp60 does not alter ATP synthase activity. However, reduction of pHi to 7.10±0.07 (*p* = 0.03) following stimulation of EC with anti-ATP synthase β-chain Ab was abrogated when EC were pre-incubated with recombinant hsp60. Thus, pHi was up-regulated to 7.29±0.06 (*p* = 0.03). These results indicate that hsp60 preserved EC from a decrease in pHi subsequent to the binding of anti-ATP synthase Abs at least when they bind to the β chain.

## Discussion

This study establishes specific interactions between ATP synthase and hsp60 on the surface of EC, based on Far WB associated with mass spectrometry. Because a single spot may contained several proteins, mass spectrometry will detect the most abundant species. We, therefore, decided to verify the specific interactions using different approaches. Co-immunoprecipitation and surface plasmon resonance analyses confirmed ATP synthase and hsp60 interaction. We determined that soluble hsp60 interacts with the 51 kDa β-subunit of ATP synthase on EC surface. In the mitochondria, hsp60 associates with at least two proteins [8]. One is the 55kDa α-subunit and the second is a 40kDa protein that has not been identified but that could be a product of the β-subunit as suggested herein in Figure 2B. These observations imply that α-subunit and β-subunit of ATP synthase would be able to associate with hsp60 in the mitochondria, whilst cell surface ATP synthase binds hsp60 with its β-subunit only. We did not observe association with the α-subunit. Whether differential conformation of F1-ATP synthase between mitochondrial and cell surface molecules might be responsible for this difference remains to be demonstrated. Our findings suggest that hsp60 might serve as a chaperon to protect ATP synthase from degradation on EC membranes. However, chaperon activity requires the presence of ATP which is lacking in our *in vitro* experiments. This aspect is thus unlikely to occur. Moreover, we can not exclude the possibility that other chaperons such as mitochondrial hsp70 may also be able to interact with ATP synthase. Furthermore, the fraction of ATP synthase that co-localized with hsp60 into lipid rafts (not shown) suggest that hsp60/ATP synthase interactions participate also in signaling, and thereby influence ATP synthase-dependent activation and proliferation of EC [25], [26]. This is consistent with other observations on extracellular ATP synthase in lipid rafts [27].

The second part of our work focused for the first time on determining the pattern of anti-ATP synthase reactivity in sera from patients containing AECAs known to be potentially pathogenic in vasculitides [28]–[30]. The frequencies of positivity were higher in patients with primary vasculitides (group I) than in those with secondary vasculitides (group II). The 3 major α-, β- and γ-subunits that constitute the F1 structure [2] were found antigenic. Intriguingly, sera from patients with primary vasculitides had a homogeneous pattern of Ab reactivity to the β-subunit of ATP synthase. By contrast, Abs from secondary vasculitides were multi-reactive. The most heterogeneous pattern was seen in sera from SLE patients with mono-, bi- and tri-reactivities. RA sera had only 2 different mono- and one bi-reactivity patterns, while Abs in the sera of pSS were homogeneous with a striking elevated frequency of mono-reactivity for the γ-subunit and 2 minor bi-reactivities. Overall, there was possiblity that patients with Abs directed to the β-subunit were those with active vasculitic disease at the time they were sampled. However, further analysis could not establish an influence of disease activity nor of medication on the Ab pattern, and reactivity with the β-subunit was not restricted to group I patients. The reasons for mono-reactivity in group I and multi-reactivity in group II sera remain largely unknown.

Modulation of ATP synthase activity was addressed in the last part of our experiments. The main function of cell surface ATP synthase appears to act as proton transport [3]. Proton flux across the membrane allows rotation of the γ-subunit, which in turn, induces modification of the α- and β-subunit conformation required for ATP synthesis [1]. When incubated in acidic pHe medium, cell surface ATP synthase is activated to preserve pHi and helps EC to survive [4]. Therefore, decreased pHi of EC observed in the presence of anti-ATP synthase Ab from patients may result from different effects. Binding of Abs to the γ-subunit might block its rotation, whilst binding to the α- or β-subunits may alter their conformational changes. Consequently, Abs inhibit ATP synthase activity and decrease pHi of EC. These observations are consistent with others based on the use of polyclonal anti-β-subunit Abs that produce intracellular acidification of EC triggering cell death [5]. However, this interpretation is not in line with other data [5], where any effect was observed with polyclonal anti-γ-subunit Abs. We suggest that inhibition of ATP synthase activity is likely dependent on specific epitope recognition rather than determined by a particular subunit binding. It is noteworthy that anti-ATP synthase mAb 7H10 directed to the α-subunit inhibits ATP synthase activity [5] whereas other anti-α-subunit clones do not [31]. Conceivably, the absence of effects observed with the positive control sera, and the differential responses observed in group I compared with group II sera (Figure 5A) might reflect disparate epitope bindings. Furthermore, piceatannol and oligomycin, inhibitors of ATP synthase [23], [24], have different effect on intracellular acidification. When EC were incubated in neutral or basic pHe, piceatannol still promoted decreases of pHi. This might be due to its ability to inhibit phosphorylation pathway [32]. Consequently, dephosphorylation of NA^+^/H^+^ transporter would be responsible for pHi diminution [33]. Whether some Abs may affect similarly ATP synthase remains to be determined.

Of interesting note is that the effects of anti-ATP synthase Abs depend on the pHe environment since no variations could be seen in EC incubated in media with neutral or basic pHe. This raises the possibility that anti-ATP synthase Abs, as recently suggested in Alzheimer's disease [34], may contribute to the pathogenicity of renal tubular acidosis that can be observed in patients with primary vasculitides [35] and in pSS patients with secondary vasculitides [36], or in SLE patients developing interstitial nephritis [37]. Furthermore, acidosis, considered as a hallmark of tumor microenvironment [3], is associated with lymphoma in patients with pSS [38]. Thus, among AECAs, anti-ATP synthase Abs should be considered as pathogenic contributors of EC deregulation. It is noteworthy that vasculitides represent heterogeneous inflammatory diseases that affect different ECs in arteries, arterioles, capillaries, veinules and veins toward major body regions [39]. Therefore, there are possibilities that in *in vivo* situations, effects of anti-ATP synthase Abs may vary from one type of ECs to another.

In addition, binding of hsp60 to ATP synthase might compete with Ab and inhibits the anti-β chain ATP synthase Ab-induced pHi down-regulation (Figure 5D). This indicates that soluble hsp60, whose level is elevated in vasculitides [21], may have protective effects. It remains to determine whether the effectiveness of the protective effect of hsp60 occurs regardless of the specificity of the anti-ATP synthase Ab. Nevertheless, at low pHe environments, this “danger” signal allows EC to maintain normal pHi owing to the preservation of a functional ATP synthase, and promotes cell survival. These effects may depend on the environment. It has thus been suggested that expression of hsp60 increases at sites predisposed to atherosclerotic lesions in response to EC stress [40]. Furthermore, arteriosclerosis can be induced or aggravated by hsp60 immunization [41], [42] implying that hsp60 can contribute to the development of cardiovascular diseases [43].

In sum, we provide new insights into the pathogenicity of AECAs in vasculitides. Recognition of cell surface ATP synthase at low pHe microenvironment contributes to intracellular acidification of EC known to induce cell death and the triggering of inflammation. However, the concomitant presence of soluble hsp60 might partly offset this deleterious response due to the specific interaction with ATP synthase. Further investigations are required to design therapeutic strategies derived from these pathogenic mechanisms.

## Materials and Methods

### Ethics statement

Ethics approval was given by the Institutional Review Board of the Brest University Hospital, of Lyon Hospital and of La Pitié Salpétrière Hospital. Use of samples from WG, CSS, MPA and PAN were approved by the Institutional Review Board of Lyon Hospital and La Pitié Salpétrière Hospital respectively, and those from SLE, RA and pSS by that of the Brest University Hospital. All participants gave their written informed consent to participate in this study.

### Cell cultures

HUVEC were prepared by digestion with 0.1% collagenase, grown to confluence [11], and passaged twice in DMEM (Gibco) with 10% fetal calf serum, 2mM glutamine and 100IU/mL polymyxin B prior to use.

The EAhy926 EC were grown in DMEM supplemented with 10% fetal calf serum, 2mM glutamine, 100 µM hypoxanthine, 0.4 µM aminopterin, 16 µM thymidine and 50mg/L gentamycin.

### Two-dimensional electrophoresis

HUVEC were detached from culture flasks with trypsin, washed and resuspended in a homogenization buffer (1M sucrose, 100mM Tris-Hcl, 100mM EDTA, 50mM MgCl_2_, 1 µM leupeptin, 1 µM pepstatin, 1 µM aprotinin). After freezing-thawing cycles, sonication, and centrifugation, membrane-enriched protein fractions were resuspended in solubilization buffer (4% CHAPS, 1% Triton X-100), centrifuged at 10,000g, and protein concentration determined using the Micro BCA protein assay kit (Pierce).

Protein extracts were resuspended in re-hydration buffer (4% CHAPS, 1% Triton X-100, 7M urea, 2M thiourea, 0.48% 3–10 biolyte, 1% tributylphosphine), and loaded onto pH 3–10 or pH 4–8 immobilized pH gradient strips (BioRad) for isoelectric focusing. After passive re-hydration, proteins were focused with a protean immunoelectrofocalisation cell (BioRad). Strips were then incubated in equilibration solutions before subjected to 10% SDS-PAGE. For the spot identifications, proteins of interest were cut out from the Coomassie blue-stained gel, digested with trypsin, desalted and deposited on 600 µm Scout MTP 384 AnchorChip (Brucker Daltonics). Peptidic fingerprints were obtained by mass spectroscopy analysis using the Ultraflex MALDI (Brucker Daltonics). The threshold for positivity was scored at 78. The detected peptide masses were then searched against Swiss-Prot dabatase.

### Co-immunoprecipitation

Cell surface proteins were cross-linked with 20 µg/ml bis(sulfo-*N*-succinimidyl) for 20 min at 4°C. After addition of 1vol of 10mM Tris, membrane-enriched fractions were obtained using a modified solubilization buffer (50mM Tris, 1% Triton X-100, 150mM NaCl). Protein solutions were pre-cleared with protein G-coated beads, and precipitated with polyclonal anti-hsp60 or anti-ATP synthase Ab-coated protein G beads (Abcam). The proteins were washed in modified solubilization buffer, and retained proteins eluted with 0.1% Triton X-100 and 0.1M triethylamin, pH 11.8 and resolved by WB.

### Western and Far Western blotting

Proteins were transferred onto PVDF membranes and probed with anti-hsp60 mAb (clone LK-1, Calbiochem) or anti-ATP synthase mAb (clone 3D5, Abcam), amplified with biotinylated anti-Ig Ab (Jackson), and developed with horseradish peroxydase (HRP)-conjugated streptavidin (Amersham). For the Far WB experiments, PVDF membranes were first incubated with 2 µg/ml recombinant hsp60 (Sigma), and then probed with anti-hsp60 mAb before development.

### Detection of cellular expression

HUVEC and EAhy926 cells were suspended in PBS, incubated with anti-ATP synthase mAb and FITC-conjugated secondary Abs, washed and analyzed in an Epics XL (Beckman Coulter) flow cytometer. Cells stained with an isotype control Ab and the FITC-conjugated Abs were enabled to set the level of positivity.

For indirect immunofluorescence staining, EC were cultured onto 10-well slides until confluence. They were incubated with anti-ATP synthase or isotype control mAb, and FITC-conjugated secondary Ab and propidium iodide, fixed in paraformaldehyde, and slides mounted in glycerol and examined with a TCS-NT confocal microscope (Leica).

### Surface plasmon resonance-based analysis

Surface plasmon resonance analyses were performed using the Biacore X system from GE Healthcare (INSERM U613, Brest). Due to the heterogeneity of the purified bovine ATP synthase complex, recombinant hsp60 was immobilized onto a carboxymethyldextran biosensor chip using amine coupling. Briefly, a continuous flow of 5 µl/min was maintained over the sensor in 10mM HEPES, 150mM NaCl, 2mM EDTA. The matrix of the chip was activated by a 7-min injection of 0.05M *N*-hydroxy-succinimide with 0.3M N-ethyl-N-(3-diethylaminopropyl) carbo-diimide, followed by a 6-min injection of recombinant hsp60 in 10mM sodium acetate, pH4.5. The procedure was completed by a 7-min exposure to ethanolamine hydrochloride to inactivate residual esters. Density of immobilized hsp60 was ∼10ng/mm^2^. Purified bovine ATP synthase was injected at a flow rate of 10 µl/min over the immobilized recombinant hsp60 (Flow Cell 2). In control experiments, another part of the same sensor was treated as above but in the absence of recombinant hsp60 (Flow Cell 1). Sensors were regenerated after each cycle by injection of a 2-µl pulse of 50mM NaOH to dissociate the analyte from the ligand. The final sensograms were calculated by substrating the signal of the control (Flow Cell 1) from that of the test (Flow Cell 2), and results expressed in resonance units.

### Patients

Group I sera comprised primary systemic vasculitides: 22 patients with Wegener's granulomatosis (WG), 14 with Churg-Strauss syndrome (CSS), 6 with polyarteritis nodosa (PAN) and 10 with microscopic polyangiitis (MPA). Group II sera comprised secondary systemic vasculitides associated with autoimmune diseases: 64 patients with systemic lupus erythematosus (SLE), 18 with primary Sjögrens' syndrome (pSS) and 20 with rheumatoid arthritis (RA). All patients are Caucasian and fulfilled the criteria for their respective disease and the sera were selected based on their positivity for AECAs [44]. Concurrently, sera from 30 laboratory staff volunteers were studied as controls. They were matched for age and sex to the two groups of patients. Thus, there were 3 women for 1 man, and their ages ranged from 23 to 66 years.

### Measurement of ATP synthase reactivity

Anti-ATP synthase autoAbs were detected using an in-house made ELISA [45]. Microtiter plates were sensitized with 0.5 µg of purified bovine ATP synthase at 5 µg/ml, blocked with PBS containing 2% BSA, incubated with 100 µL of serum diluted 1∶50 in 1% BSA and developed with alkaline phosphatase-conjugated anti-human IgG (Zymed). Optical density (OD) of control wells without ATP synthase were subtracted from the OD of ATP synthase-coated wells. The mean+2 standard deviations (SDs) of the OD values in negative control sera was taken as the threshold for positivity.

Reactivity was also determined by WB to evaluate the recognition of ATP synthase subunits. The protein complex was subjected to 10% SDS-PAGE, transferred onto PVDF membranes saturated with PBS containing 5% milk proteins, and probed with sera diluted 1∶100 in 1% milk proteins. Bound autoAbs were revealed with biotinylated-anti-human IgG Ab (Jackson) and developed with HRP-streptavidin. After densitometry analyses performed with Quantity-One® software (BioRad) for each α-, β- and γ-subunit, the mean+2 SD of the OD values for the control sera were the threshold for positivities.

### Assessment of intracellular pH and ATP synthase function

pHi was measured by flow cytometry. Briefly, EAhy926 cells were suspended in HBSS supplemented with 0.32 µM BCECF (Invitrogen). Cells were then incubated at 37°C to allow BCECF to be hydrolyzed by intracellular esterase into pH-dependent fluorescent probe and analyzed in an Epics XL flow cytometer. Excitation at wavelength 488nm led to green and red fluorescences that were selected using a 525nm and a 640nm bandpass filter, respectively. While emission intensity at 525nm is stable that at 640nm decreases with pHi. Therefore, 525/640 nm ratio that increases linearly with pH was considered as an appropriate tool for the evaluation of ATP synthase activity [46]. To ascertain the reproducibility of the method, calibration curves were constructed for each experiment from BCECF-labelled cells suspended in media with different pHe, and in the presence of 40 µM nigericin (Sigma), an ionophore that exchanges K^+^ and H^+^ across cellular membranes. In these conditions, pHi measured corresponds to pHe [22].

EAhy926 cells labelled with BCECF were incubated 1∶20 with 100 µL of serum, or with 50 µg/mL anti-ATP synthase mAb (clone 4.3E8.D10, Sigma-Aldrich) for 1 h at 4°C. The cells were then washed in medium with appropriate pHe and incubated in the same medium for 20 minutes at 37°C before flow cytometry analysis. Modulation of pHi was then evaluated. Positive control was performed using BCECF-loaded cells suspended in medium with different pHe and in the presence of 80 µM piceatannol (Sigma), a tetrahydroxystilbene that inhibits specifically ATP synthase [23].

In some experiments, sera were pre-depleted of their anti-ATP synthase reactivity by overnight incubation at 4°C in ATP synthase-coated microplates. Finally, in some experiments, 5.10^6^ BCECF-loaded EC were incubated with 8 µg recombinant hsp60 at 80 µg/ml, before suspension with diluted sera.

### Statistical analysis

All data were expressed as the mean±SD. Statistical analysis used chi-square or Fisher's exact test for comparisons of percentages. Mean quantitative values were compared using the Wilcoxon or Mann and Whitney U-test. Significance was assessed at *p* = 0.05.
